# Cognitive Behavioral Therapy for Paroxysmal Atrial Fibrillation: Heart Rate Variability, Physical Activity, and Sleep

**DOI:** 10.1016/j.jacadv.2024.101289

**Published:** 2024-09-27

**Authors:** H. Skúladóttir, J. Särnholm, E. Ólafsdóttir, E.S. Arnardóttir, K. Hoppe, M. Bottai, B. Ljótsson, F. Braunschweig

**Affiliations:** a Department of Cardiology, Karolinska University Hospital, Stockholm, Sweden; b Division of Psychology, Department of Clinical Neuroscience, Karolinska Institutet, Stockholm, Sweden; c University Sleep Institute, School of Technology, Reykjavik University, Reykjavik, Iceland; d Philips Ambulatory Monitoring and Diagnostics, Copenhagen, Denmark; e Institute for Environmental Medicine, Karolinska Institutet, Stockholm, Sweden

**Keywords:** anxiety, insomnia, Patch-Holter, steps, symptomatic

## Abstract

**Background:**

Cognitive behavioral therapy for symptom preoccupation in paroxysmal atrial fibrillation (AF-CBT) significantly improved AF-specific quality of life in a recent trial. To what extent this may this may be associated with changes in physiological parameters is yet to be determined.

**Objectives:**

The purpose of this study was to assess the effects of AF-CBT on heart rate variability (HRV), physical activity, and sleep.

**Methods:**

Patients with symptomatic paroxysmal AF on optimal medical therapy (mean ± standard deviation 65.4 ± 8.3 years, 58% females, 61% on beta-blockers) were randomized to a 10-week online AF-CBT (n = 65) or AF education (n = 62). AF-CBT was therapist-led and focused on social and physical avoidance. A 5-day Patch-Holter with an integrated accelerometer was applied at baseline, post-treatment, and at 3-month follow-up to assess AF burden, HRV, physical activity, and sleep duration. Subjective assessments were made by the International Physical Activity (IPAQ) and Insomnia Severity Index questionnaires.

**Results:**

At baseline, participants walked on average 8040 ± 2600 steps/day and slept 8.0 ± 1.1 hours. Objective and subjective physical activity and sleep duration remained unchanged after treatment, with no significant difference between the groups. The Insomnia Severity Index score went from subclinical insomnia (12.2 ± 6.7) to near normal values (8.1 ± 5.6), a significant change compared to controls (*P* = 0.032). No changes were found in AF burden or HRV indices at rest during the day or at night.

**Conclusions:**

In this select cohort, AF-CBT decreased insomnia severity but had no impact on HRV or physical activity. Thus, AF-CBT may operate through psychological and behavioral factors that are not targeted by current medical and lifestyle interventions.

Paroxysmal atrial fibrillation (AF) is characterized by episodes of an abrupt and unexpected nature that can lead to significant psychological distress and a markedly reduced quality of life (QoL).^1^^,^^2^

Medical treatment for AF aims to control heart rate or rhythm and includes antiarrhythmic drugs and invasive ablations. Rhythm control is, however, not always effective and is associated with potentially serious side effects.^3^ Resources are also limited, and many patients are either unsuitable for or unable to access AF ablations. At the same time, the prevalence of AF continues to rise due to global demographic changes and the obesity pandemic.^4^

Managing modifiable risk factors can decrease the arrhythmia burden and enhance QoL, and lifestyle interventions are now an integral part of evidence-based AF care.^5^ Physical inactivity, alcohol consumption, and sleep disorders are indeed associated with automatic nervous system (ANS) dysfunction, a key element in AF pathophysiology.^6^ Heart rate variability (HRV) can be helpful in assessing ANS dysfunction in patients with paroxysmal AF, and studies have shown that HRV indices can improve after an invasive ablation or lifestyle improvements.^7^^,^^8^

Anxiety is another factor that is strongly related to reduced HRV,^9^ and the link between physiological and psychological stress in AF might even be bidirectional, with emerging research suggesting that “stress begets AF”.^10^ Furthermore, psychological distress can lead to negative behaviors, such as physical and social avoidance and poor sleep, which can directly impair QoL.^11^, ^12^, ^13^

We have devised a novel cognitive behavioral therapy that targets symptom preoccupation in paroxysmal AF (AF-CBT) and shown that it significantly improved disease-specific QoL in a randomized controlled trial.^14^, ^15^, ^16^ AF-CBT is delivered online and guided by a clinical psychologist and includes structured exposure to feared and avoided activities. It is, therefore, plausible that it leads to beneficial changes in physiological lifestyle-related factors. The aim of this study was to determine changes in HRV and objective and subjective measures of physical activity and sleep in subjects randomized to 10-week online AF-CBT compared to controls that received AF education only.

## Methods

### Study design

This study was based on data from a randomized controlled trial evaluating the effect of CBT on disease-specific QoL in patients with symptomatic paroxysmal AF, performed at the Karolinska University Hospital in Stockholm, Sweden.^16^ The trial was approved by the Regional Ethics Review Board in Stockholm (no. 2017/1882-31/2), preregistered at ClinicalTrials.gov (NCT03378349), and conducted in accordance with the Declaration of Helsinki and all relevant regulations. The authors vouch for the completeness and accuracy of the data and fidelity to the protocol.

### Trial protocol

A detailed study protocol and a recruitment flowchart have been published previously.^16^ In brief, patients with electrocardiography (ECG)-documented paroxysmal AF, with at least one subjective episode per month, and AF-related symptoms corresponding to European Heart Rhythm Association class ≥IIb ^17^ were included after a referral from cardiology clinics in the Stockholm area or self-referral in response to an advertisement in local papers. All patients were assessed at the cardiology clinic by the study nurse (E.Ó.), who reviewed their medical history and medical records and acquired blood pressure, a 12-lead ECG, weight, and height. An echocardiogram was performed on-site if not taken within the previous 12 months. All medical information was reviewed by the study cardiologist (H.S.) to ensure that they were on optimal medical therapy and that all medical inclusion and exclusion criteria were fulfilled. All participants were assessed through a structured clinical interview conducted by a licensed psychologist. The interview adhered to the Diagnostic and Statistical Manual of Mental Disorders, Fifth Edition criteria and included open-ended questions to determine if there was a significant clinical level of psychiatric comorbidity. Exclusion criteria were heart failure with a left ventricular ejection fraction ≤35% or significant valvular disease, AF ablation performed within 3 months or planned AF ablation, any other severe medical illness, medical restriction to physical exercise, severe psychiatric disorder (eg, major depression), alcohol dependency and concurrent psychological treatment or participation in any other interventional or rehabilitation program that could interfere with the treatment.

### Intervention

**Figure 1:**
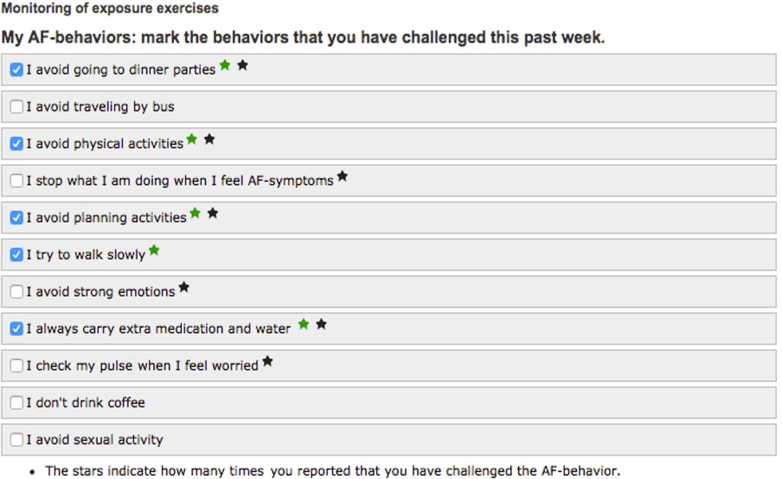
**Online Exposure Exercises in Cognitive Behavior Therapy for Paroxysmal Atrial Fibrillation** An example of an individual list of exercises performed in cognitive behavior therapy for symptom preoccupation in paroxysmal atrial fibrillation. AF = atrial fibrillation.

AF-CBT was delivered online and targeted avoidance behavior, fear of triggering AF episodes, and hypervigilance toward (perceived) cardiac symptoms. It consisted of systematic exposure to feared and avoided activities, social situations, or physical sensations similar to AF symptoms. Excessive symptom-controlling behavior, such as repeated pulse checking, was discouraged. The treatment was therapist-led and comprised six text-based modules and exposure exercises over 10 weeks. The exercises were individualized by creating a specific list of AF-related avoidance and control behaviors that each patient would challenge during the treatment (Figure 1).

The control group received standardized education on AF pathophysiology, symptoms, and medical therapy.^18^ Lifestyle factors, such as weight control and physical activity, were also addressed, and moderate exercise, such as brisk walks for 3.5 hours per week, was recommended. After a 3-month follow-up period, the control group was switched to receive treatment.

### Subjective outcome measures

The seven-item Insomnia Severity Index Questionnaire assessed insomnia.^19^ Participants also reported time spent doing moderate and vigorous exercise on the short International Physical Activity Questionnaire (IPAQ) form.^20^ The primary outcome of the main trial was AF-specific QoL, as assessed by the Atrial Fibrillation Effect on Quality of Life Questionnaire.^21^ The Cardiac Anxiety Questionnaire, an 18-item form, was used to assess cardiac-related fear, avoidance behavior, and attention to cardiac-related symptoms.^22^

### Objective outcome measures

A 2-channel Patch-Holter (ePatch, Philips) was attached at the baseline visit and then sent by mail for follow-up measurements, each for a total of 5 days of recording. The study devices were specifically manufactured with an integrated 3-axis accelerometer with a sampling rate of 32 Hz to provide synchronous information on steps and posture.

Outcome measures were prospectively assessed at baseline, after treatment, and 3 months post-treatment, and all analyses were performed after the trial was completed.

### Signal processing and analysis

All Holter recordings were analyzed in a blinded fashion using designated HRV software (Cardiscope, ANALYTICS, professional edition, version 1.3.230) according to standards and norms.^23^ Noise, artifacts, and aberrant beats were filtered out, and AF episodes and beats deviating more than 20% from the previous RR interval were excluded, leaving only normal sinus beats and their intervals (NN intervals) for HRV analysis. Each 5-day recording was reviewed by the study cardiologist (H.S.) and divided manually into day and night (sleep time) based on posture, activity, heart rate, and HRV. A predefined hour in the afternoon, when the patient was at relative rest, was also manually selected for HRV analysis.

The following HRV parameters were calculated in the time domain: standard deviation from the mean interval between two consecutive normal beats and the root mean square of successive differences in NN interval, which captures short-term vagally mediated beat-to-beat differences. The spectral components of HRV, the high-frequency band (HF: 0.15-0.4 Hz, reflecting vagally mediated respiratory sinus arrhythmia), and low-frequency band (LF: 0.04-0.15 Hz, reflecting baroreceptor activity) were separated by Fourier transformation. LF and HF are expressed as indexed values (LF_i_, HF_i_), that is, the average of all valid overlapping 5-minute episodes. The spread of NN intervals was depicted in a scattergram (Poincaré plot), with each interval plotted against the preceding one and described quantitatively as the standard deviation of the plot perpendicular to the oblique line of identity (SD1).^23^

### Statistical analysis

Statistical analyses were performed using Stata, version 14.2 (StataCorp) and R, version 4.1.1 (R Project for Statistical Computing) by authors (H.S. and M.B.). The outcomes were assessed with linear generalized estimating equations. The dependent variables were time (as a numeric variable coded as 0 = baseline, 1 = post-treatment, and 2 = 3-month follow-up), group (as a binary indicator), and the interaction between time and group. No data imputation was performed as missing data were negligible. The linear generalized estimating equations used an identity link function. The standard errors of the regression coefficients were estimated with a sandwich cluster-robust estimator assuming an exchangeable working correlation structure.

All results are expressed as mean ± SD or a 95% CI. A *P* value <0.05 was considered statistically significant. The reliability over time of the HRV indices was assessed with the intraclass correlation coefficient (ICC). An ICC between 0.50 and 0.75 is generally considered to indicate moderate reliability, values between 0.75 and 0.90 good reliability, and values greater than 0.90 excellent reliability.^24^

## Results

### Participants

The study included 127 patients, of which 58% were females. The average age was 65.4 ± 8.3 years, and the mean body mass index was 27.1 ± 5.5 kg/m^2^. One hundred nineteen patients (94%) completed the 3-month follow-up. Baseline characteristics are shown in Table 1. More comprehensive information on participant characteristics has been published elsewhere.^16^

**Table 1:** Participants’ Characteristics at Baseline

	All (N = 127)	AF-CBT (n = 65)	Control (n = 62)
Age (y)	65.4 ± 8.3	65.1 ± 8.4	65.7 ± 7.5
Females	74 (58)	38 (58)	36 (58)
Body mass index (kg/m^2^)	27.1 ± 5.5	27.1 ± 6.7	27.2 ± 3.9
Previous ablation(s)	13 (10)	6 (9)	7 (11)
CHA_2_DS_2_-VASc score	1.9 ± 1.2	1.8 ± 1.2	2.0 ± 1.2
OSA	25 (20)	10 (15)	15 (24)
CPAP/OA	14 (11)	4 (6)	10 (16)
Physical therapy	20 (16)	11 (17)	9 (15)
AF burden	5.1%	5.3%	5.0%
Any anxiety disorder	31 (24)	16 (25)	15 (24)
Depressed mood	37 (29)	21 (32)	16 (26)
Antiarrhythmics	32 (25)	17 (26)	15 (24)
Beta-blockers	78 (61)	39 (60)	39 (63)
Beta-blockers “as needed”	25 (20)	15 (23)	10 (16)
Anxiolytics “as needed”	7 (6)	4 (6)	3 (5)
Sleep medication	17 (13)	11 (17)	6 (10)

Values are mean ± SD or n (%).

AF-CBT = cognitive behavioral therapy for atrial fibrillation; CHA_2_DS_2_-VASc = congestive heart failure, hypertension, age, diabetes mellitus, prior stroke or TIA or thromboembolism, vascular disease, age, and sex category risk score; CPAP = continuous positive airway pressure; OA = oral appliance; OSA = obstructive sleep apnea.

### Previously reported quality of life and cardiac anxiety

The effects of AF-CBT on QoL and cardiac anxiety have been published previously.^16^ In brief, the primary outcome AF-specific QoL increased from 62.4 ± 14.3 to 83.7 ± 13.8 points on the Atrial Fibrillation Effect on Quality of Life Questionnaire at the 3-month follow-up, corresponding to a change from moderate to mild impairment, with a relative difference compared to the control group of 15.0 points (*P* < 0.001, score range: 1-100). Cardiac anxiety was significantly reduced from clinically significant levels to subclinical levels (31.6 ± 8.5-16.6 ± 9.5 points, score range: 0-72), with a relative difference of 9.9 points (*P* < 0.001).

### Heart rate variability

The average resting heart rate was 67.6 ± 9.7 beats/minute during the day and 57.5 ± 6.7 beats/minute at night in all subjects, which was not affected by the AF-CBT. Time and frequency domain HRV, at rest in the daytime or at night, also remained unchanged (Table 2). Both groups showed a significant intra-individual correlation in heart rate and HRV indices over time (Table 2). Two separate post hoc subgroup analyses on patients fulfilling diagnostic criteria for any anxiety disorder (24%) and patients who were not taking beta-blockers on a regular basis (39%) showed no significant change in HRV parameters with AF-CBT (*P* values for all HRV indices >0.25).

**Table 2:** Effects of Cognitive Behavioral Therapy for Paroxysmal Atrial Fibrillation on Heart Rate Variability

	AF-CBT (n = 65)			Control (n = 62)			*P* Value	ICC
	Baseline	Post-Treatment	3-Month Follow-Up	Baseline	Post-Treatment	3-Month Follow-Up		
HR day (beats/min)	68.5 (66.4-70.6)	67.7 (65.5-69.8)	68.2 (65.8-70.6)	66.7 (64.3-69.1)	66.8 (64.7-69.0)	66.1 (63.8-68.3)	0.601	0.602
HR night (beats/min)	58.4 (56.9-59.8)	58.3 (56.8-59.9)	58.3 (56.7-59.9)	56.6 (54.8-58.3)	56.5 (54.8-58.1)	56.7 (55.0-58.3)	0.956	0.774
SDNN day (ms)	78.2 (72.5-84.0)	78.3 (72.3-84.4)	77.8 (71.5-84.1)	83.4 (76.7-90.2)	83.4 (76.8-90.0)	83.1 (76.1-90.1)	0.998	0.471
SDNN night (ms)	84.0 (77.1-90.9)	83.1 (76.8-89.5)	84.0 (77.7-90.3)	92.4 (85.0-99.8)	92.5 (85.2-99.8)	92.8 (85.7-99.8)	0.940	0.757
RMSSD day (ms)	34.0 (30.9-37.0)	34.1 (30.5-37.6)	33.9 (29.8-38.0)	33.7 (30.2-37.3)	34.1 (30.4-37.9)	34.3 (30.6-38.0)	0.943	0.612
RMSSD night(ms)	37.7 (32.7-42.6)	37.9 (33.3-42.6)	36.6 (32.6-40.7)	39.9 (35.1-44.6)	39.8 (35.2-44.3)	39.7 (35.4-44.1)	0.815	0.801
HFi day (ms^2^)	367.0 (291.8-442.0)	352.7 (272.7-432.6)	379.5 (266.6-492.3)	367.1 (274.3-459.8)	360.3 (269.5-451.2)	391.9 (301.0-482.8)	0.966	0.569
HFi night (ms^2^)	532.5 (382.1-682.9)	549.1 (396.4-701.8)	502.4 (371.7-633.0)	537.6 (416.1-659.1)	537.7 (427.8-647.7)	541.5 (438.0-644.9)	0.604	0.773
LFi day (ms^2^)	722.1 (539.6-904.7)	846.5 (543.8-1149.1)	742.4 (501.2-983.6)	639.7 (524.0-755.4)	644.1 (505.3-782.9)	674.6 (546.2-803.0)	0.668	0.618
LFi night (ms^2^)	863.7 (653.4-1073.9)	1073.0 (746.0-1400.0)	876.9 (645.8-1108.1)	1107.1 (823.1-1391.1)	1129.9 (824.8-1435.0)	1047.6 (793.9-1301.2)	0.409	0.771
SD1 day (ms)	24.0 (21.8-26.2)	24.1 (21.6-26.7)	23.9 (21.0-26.8)	23.9 (21.4-26.4)	24.1 (21.5-26.8)	24.2 (21.6-26.9)	0.946	0.614
SD1 night (ms)	26.6 (23.1-30.1)	26.8 (23.5-30.1)	25.9 (23.0-28.8)	28.2 (24.8-31.5)	28.1 (24.9-31.3)	28.0 (25.1-31.1)	0.816	0.802

Values are mean (95% CI). *P* values indicate different trajectories between the treatment and control groups. Daytime values are taken over a predefined hour in the afternoon when the patient is at relative rest. Changes from baseline to 3-month follow-up were assessed by linear generalized estimating equations.

HFi = high-frequency band, indexed; HR = heart rate; ICC = intraclass coefficient; LFi = low-frequency band, indexed; RMSSD = root mean square of successive differences between normal heartbeats; SDNN = standard deviation of normal-to-normal beat intervals; SD1 = standard deviation perpendicular to the line of identity on the heart rate variability scattergram; other abbreviation as in Table 1.

As previously reported, the baseline AF burden on the 5-day Holter was, on average, 5.3% in the treatment group and 5.0% in the control group, which increased to 7.3% and 9.2%, respectively, at the 3-month follow-up with no difference between the groups (*P* = 0.59).^16^

### Physical activity

**Figure 2:**
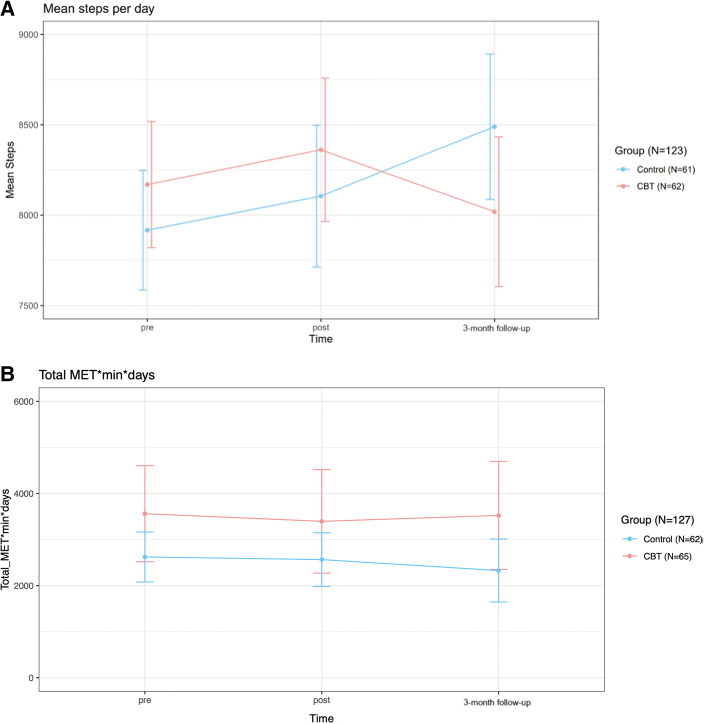
**Changes in Physical Activity Over the Study Period** (A) Objective changes in physical activity over the study period. (B) Subjective changes in moderate and vigorous activity over the study period. Steps/day were measured by a 5-day patch-holter with an integrated accelerometer at pre-, post-, and 3-month follow-up. The short International Physical Activity Questionnaire (IPAQ) was used for self-rated assessments. Values represent means, and vertical lines indicate 95% CI. CBT = cognitive behavioral therapy; MET = metabolic equivalent.

Physical activity did not increase from an average of 8040 ± 2600 objectively measured steps/day in all subjects at baseline, and there was no significant difference between the groups over time (*P* = 0.71) (Figure 2A). Likewise, the participants did not report an increase in moderate or vigorous activity on the IPAQ questionnaire, with no difference between the groups over time (*P* = 0.77) (Figure 2B).

### Sleep

**Figure 3:**
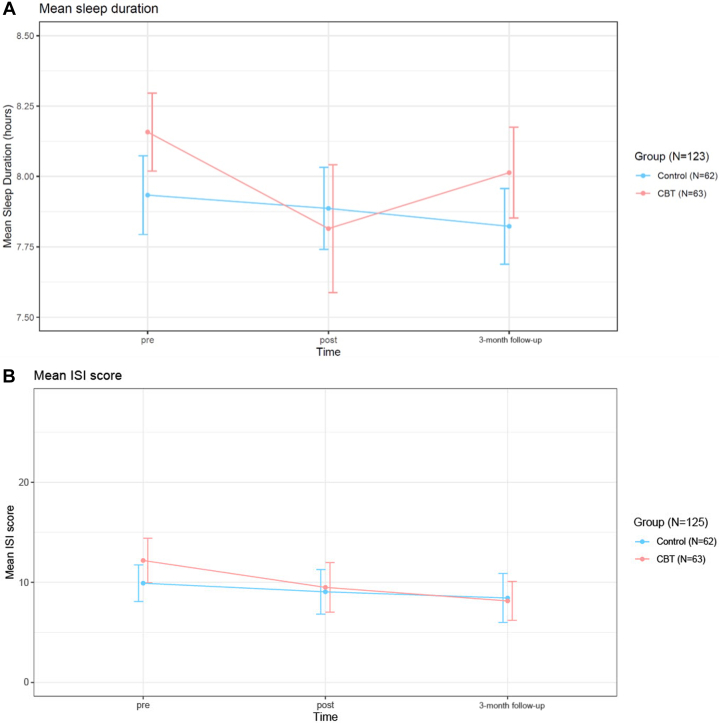
**Changes in Sleep Duration and Insomnia Over the Study Period** (A) Objective changes in sleep duration over the study period. (B) Subjective changes in insomnia severity over the study period. Sleep Duration was measured by a 5-day patch-holter with an integrated accelerometer at pre-, post-, and 3-month follow-up. The insomnia severity index (ISI) questionnaire was used for self-rated assessments. Values represent means, and vertical lines indicate 95% CI. Abbreviation as in Figure 2.

The objective measure of sleep showed that study participants slept for 8.0 ± 1.1 hours at baseline, with approximately two-thirds (82/123) falling within a 7–9-hour range. AF-CBT did not affect sleep duration, and there was no difference between the two groups over time (*P* = 0.57) (Figure 3A). Insomnia severity, as assessed by the Insomnia Severity Index, changed from 12.2 ± 6.7 U at baseline in the CBT group to 8.1 ± 5.6 at the 3-month follow-up (subthreshold insomnia scores 8-14), which was significant compared to the control group (*P* = 0.032) (Figure 3B).

## Discussion

### Principal findings

We have previously demonstrated that exposure-based online CBT significantly improves disease-specific QoL in patients with symptomatic paroxysmal AF.^16^ This study assessed the impact of AF-CBT on a series of relevant physiological variables, including HRV, physical activity, and sleep, measured both subjectively and objectively. The main finding is that these variables remained unchanged, except for insomnia severity, which improved among patients in the AF-CBT group (Central Illustration).

**Central Illustration:**
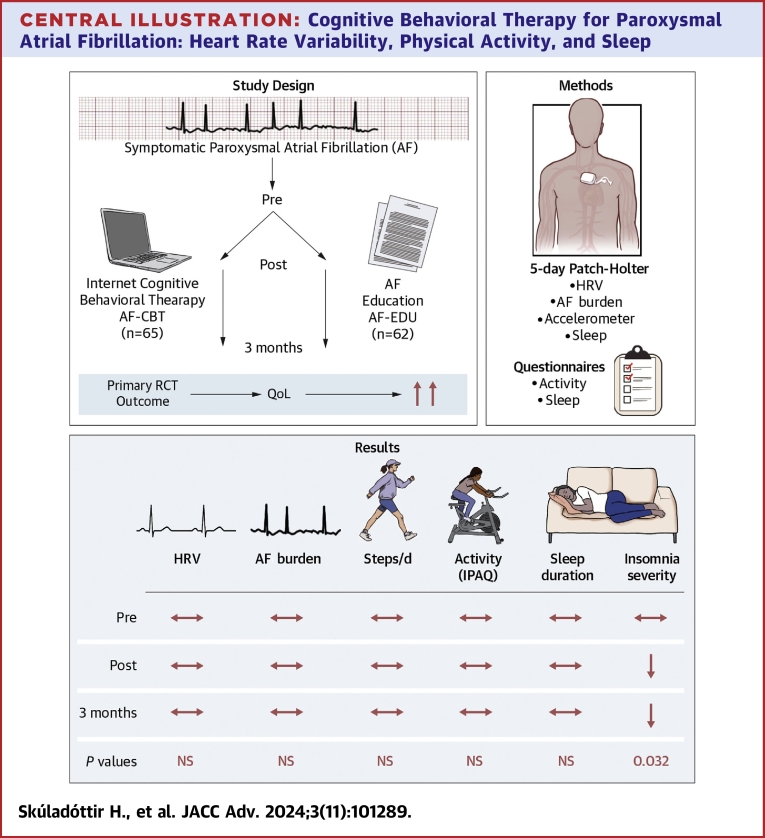
**Cognitive Behavioral Therapy for Paroxysmal Atrial Fibrillation: Heart Rate Variability, Physical Activity, and Sleep** This randomized controlled trial (RCT) substudy evaluated the effects of cognitive behavioral therapy that targets physical and social avoidance in paroxysmal atrial fibrillation on physical activity, sleep, and heart rate variability (HRV). The study cohort proved to be relatively active at baseline with 8040 ± 2600 steps/day and an average sleep duration of 8.0 ± 1.1 hours, which remained unchanged after treatment in both groups. Likewise, no changes were found in reported physical activity on the international physical activity questionnaire (IPAQ) or HRV Indices in both groups. AF-CBT has been shown to improve disease-specific quality of life (QoL), and the treatment group also demonstrated a significant improvement in insomnia severity as compared to the control group that received AF education only (*P* = 0.032), underlining the importance of subjective measures in symptomatic AF. AF-CBT = cognitive behavioral therapy for symptom preoccupation in paroxysmal atrial fibrillation.

### Comparison with other studies

That HRV remained unchanged may seem unexpected as improved QoL, in the main trial, was accompanied by significant improvements in cardiac anxiety. The absence of measurable changes in our study is paralleled by findings in recent literature. In patients with agoraphobia, measures of HRV at rest did not change despite improvements in anxiety after exposure-based CBT.^25^ A meta-analysis comparing the impact of psychotropic medication on HRV in different mental diseases showed that resting HRV did not change in patients with anxiety disorders.^26^ A third study on exposure-based CBT alone or in combination with sertraline in patients with panic disorder demonstrated a significant reduction in HR and an increase in HRV but only in the CBT-alone group.^27^ In our study, only 24% fulfilled diagnostic criteria for anxiety disorders, and a subgroup analysis did not reveal any change in HRV in this group either (data not shown). We chose not to have strict psychological inclusion criteria and excluded patients with severe psychiatric disorders. The participants were simply “troubled by” their AF symptoms, although they did not necessarily affect daily activities (ie, European Heart Rhythm Association symptom scale ≥2b).^17^ This was also reflected in the relatively low AF burden (5.1% at baseline), yielding a clear discrepancy between arrhythmia burden and QoL impairment. During the study, the AF burden showed a trend toward increase with no significant difference between the trial groups. This may reflect the natural course of AF and is also consistent with the lack of physiological responses to CBT. In contrast, if AF-CBT improved autonomic balance, as measured by HRV, that might have inferred a reduction in arrhythmia burden.

### Psychological and physiological distress

The lack of changes in HRV supports our evolving understanding of AF-related psychological impairment, which is in many ways different from traditional anxiety disorders. It can be conceptualized as a disease-specific maladaptive behavioral response in which the patient unsuccessfully tries to control perceived arrhythmia triggers, creating a vicious cycle of symptom preoccupation and avoidance.^11^^,^^14^ AF-CBT aims to break this cycle and appears to enable people to enjoy various physical and social activities without affecting physiological stress. A crucial aspect of successful exposure-based CBT is that patients continuously challenge their fears and confront avoided situations. Successful therapy does not always mean that all discomfort or sympathetic activation is cured or gone, but that the patients continue to participate in their daily lives and manage their AF (discomfort or feared stimuli) via behavioral change. AF patients can also be physically active but with heightened hypervigilance and distress, where successful therapy reduces cardiac anxiety and improves QoL while maintaining the same activity levels.

### Physical activity and sleep

The participants were relatively active, with approximately 8000 steps, and sleep duration was 8 hours on average, both in accordance with general recommendations and goals.^28^^,^^29^ Lack of significant changes in physical activity and sleep duration thus most likely reflects a ceiling effect, and although a change from a sedentary lifestyle can improve psychological well-being,^30^ this relationship becomes more obscure at higher activity levels. It is conceivable that psychological distress might even make people more prone to “pace the floor” and/or be extra careful to follow health recommendations. AF-CBT does not target insomnia specifically, but our results are in line with other studies showing a strong correlation between improvements in psychological well-being and insomnia severity.^31^

### Potential clinical value of HRV analyses in AF care

Consumer wearable devices that track advanced health metrics, including HRV by optical pulse sensors, have become widely used in the general population. Pulse sensors are, however, unreliable for rhythm analysis, and consumer wearable devices require patient interaction to record a single-lead ECG.^32^ We used a diagnostic Holter device with an integrated accelerometer to assess rhythm and resting ANS physiology and could demonstrate a significant intra-individual correlation in HRV indices over time (excluding AF episodes and aberrant beats).^33^ Thus, we have shown that it is feasible to gather combined data on rhythm, steps, sleep duration, and HRV, and we suggest that this approach might be valuable in integrated AF care as it might provide feedback on modifiable risk factors. However, it should be considered that HRV is not a perfect tool to assess the ANS. The low-frequency domain reflects both arms, the sympathetic response, and the vagal tone, whereas the high-frequency domain and the time domains (root mean square of successive differences in NN interval in particular) are more of a measure of pure vagal tone.^23^^,^^34^ The complexity of the heart, mind, and the autonomic nervous system that links those two cannot be overstated.

### Study strengths and limitations

The study's main strengths were thorough cardiac and psychological assessments and the use of subjective and objective measurements. However, some important limitations exist. The sample size was relatively small, implying a risk for type II statistical error. Of note, more than half (61%) of the participants were on beta-blockers, which could potentially ameliorate the effects of psychological stress on HRV. A subgroup analysis of those who were not taking beta-blockers on a regular basis (39%) showed similar results with no change after AF-CBT (data not shown).

Furthermore, the percentage of patients on beta-blockers in our study likely reflects the real world, as beta-blockers are widely used in patients with symptomatic paroxysmal AF.

It is possible that the participants were more active while wearing the 5-day Patch-Holter and an extended recording could have provided a more accurate result.

The study participants were partly self-referred and had a relatively low body mass index, which suggests they may not represent the average AF patient. However, this can be considered an advantage for our study, as we aimed to treat behavioral factors that are unlikely to be attainable by current lifestyle interventions. It should be emphasized that many factors, particularly physical activity and sleep, can directly affect HRV. As we did not observe any change in physical activity or sleep duration, our results show that a decrease in cardiac anxiety to subclinical levels did not affect HRV. However, we cannot exclude the possibility of a ceiling effect, although direct comparison with age-specific reference values is difficult as there is a relatively wide range, and most published data are either derived from ≤5-minute ECG recordings in a controlled laboratory setting or 24-hour Holter recordings.^23^^,^^35^, ^36^, ^37^

Polysomnography is the gold standard for sleep measurements and infers a visual assessment of an electroencephalogram and other physiological signals, including an ECG. A 3-axis accelerometer is an accepted alternative for measuring sleep duration in non-laboratory settings (so-called “free-living”), with the important caveat of being unable to identify motionless wake.^38^^,^^39^ To reduce the risk of overestimating sleep duration, we made an integrated visual assessment of the 3-axis accelerometer (posture and activity) and the ECG signal (reduced HR and the distinct appearance of an HF band reflecting vagal tone),^40^ but this approach needs to be validated against polysomnography.^41^

## Conclusions

AF-CBT improved insomnia severity but did not affect physical activity or HRV in the study cohort. These findings emphasize the importance of subjective psychological, behavioral, and social factors in symptomatic AF, and we propose that exposure-based CBT might fill a void beyond medical treatment and lifestyle interventions in AF care.

PERSPECTIVES **COMPETENCY IN PATIENT CARE AND PROCEDURAL SKILLS:** Our findings support that CBT for paroxysmal AF might become an integral part of comprehensive AF care models. The lack of effect on physiological variables emphasizes the importance of psychological and behavioral factors that can be successfully targeted to improve the well-being of a large population that is troubled by AF symptoms.

**TRANSLATIONAL OUTLOOK:** Further research is necessary to assess AF-CBT in a broader population, perceivably with adjustments to meet the needs of those with suboptimal lifestyles.

## Funding support and author disclosures

This study was supported by grants from the Swedish Research Council (2016-013792016-01379); Region Stockholm; the ALF project (20160260), Stockholm, Sweden; Mats Kleberg’s Foundation (2015-00088), Stockholm, Sweden; and Karolinska University Hospital, Stockholm, Sweden. None of the funding bodies had any influence on the study design, implementation, data analysis, or interpretation. Dr Skúladóttir has received lecture fees from Novo Nordisk and AstraZeneca. Dr Ljótsson has authored a self-help book based on exposure-based cognitive behavior therapy for health anxiety that is available in the public marketplace. Dr Arnardóttir has received honoraria from Nox Medical, ResMed, Jazz Pharmaceuticals, Linde Healthcare, Wink Sleep, Apnimed, and Vistor, as well as being a member of the Philips Sleep Medicine and Innovation Medical Advisory Board. All other authors have reported that they have no relationships relevant to the contents of this paper to disclose.
